# Advances in genomic tools for plant breeding: harnessing DNA molecular markers, genomic selection, and genome editing

**DOI:** 10.1186/s40659-024-00562-6

**Published:** 2024-11-07

**Authors:** Rahul Kumar, Sankar Prasad Das, Burhan Uddin Choudhury, Amit Kumar, Nitish Ranjan Prakash, Ramlakhan Verma, Mridul Chakraborti, Ayam Gangarani Devi, Bijoya Bhattacharjee, Rekha Das, Bapi Das, H. Lembisana Devi, Biswajit Das, Santoshi Rawat, Vinay Kumar Mishra

**Affiliations:** 1 ICAR Research Complex for NEH Region, Tripura Centre, Lembucherra, Agartala, 799210 Tripura India; 2 ICAR-National Research Centre for Orchids, Pakyong, Sikkim India; 3 ICAR Research Complex for NEH Region, Umiam, 793103 Meghalaya India; 4 ICAR-Central Soil Salinity Research Institute, Karnal, India; 5 ICAR-National Rice Research Institute, Cuttack, 753006 Odisha India; 6 ICAR –Krishi Vigyan Kendra, Tamenglong District, Manipur, India; 7 Department of Food Science and Technology, College of Agriculture, G.B.P.U.A.&T., Pantnagar, India

**Keywords:** Molecular markers, Genomic selection, CRISPR-Cas9, Genomic tools, Genomic resources, Plant breeding

## Abstract

Conventional pre-genomics breeding methodologies have significantly improved crop yields since the mid-twentieth century. Genomics provides breeders with advanced tools for whole-genome study, enabling a direct genotype–phenotype analysis. This shift has led to precise and efficient crop development through genomics-based approaches, including molecular markers, genomic selection, and genome editing. Molecular markers, such as SNPs, are crucial for identifying genomic regions linked to important traits, enhancing breeding accuracy and efficiency. Genomic resources viz. genetic markers, reference genomes, sequence and protein databases, transcriptomes, and gene expression profiles, are vital in plant breeding and aid in the identification of key traits, understanding genetic diversity, assist in genomic mapping, support marker-assisted selection and speeding up breeding programs. Advanced techniques like CRISPR/Cas9 allow precise gene modification, accelerating breeding processes. Key techniques like Genome-Wide Association study (GWAS), Marker-Assisted Selection (MAS), and Genomic Selection (GS) enable precise trait selection and prediction of breeding outcomes, improving crop yield, disease resistance, and stress tolerance. These tools are handy for complex traits influenced by multiple genes and environmental factors. This paper explores new genomic technologies like molecular markers, genomic selection, and genome editing for plant breeding showcasing their impact on developing new plant varieties.

## Background

Since plant domestication around 10,000 years ago, plant breeding has successfully developed crops and varieties essential to modern society, consistently defying Malthusian predictions [44]. Traditional pre-genomics breeding methods have resulted in modern cultivars, significantly increasing the yields of major crops since the mid-twentieth century. Today, genomics offers breeders advanced tools and techniques for whole-genome analysis, representing a significant shift by enabling direct examination of the genotype and its connection to the phenotype [166, 167]**.** This new era of crop development leverages genomics-based approaches, such as molecular markers, genomic selection, and genome editing tools, for precise and efficient improvements [166, 167]. Gene position or genomic regions that regulate important traits in plants are discovered using molecular markers. Markers are typically categorized into two main groups: classical and molecular markers. The limitations associated with phenotype-based markers prompted the development of direct DNA-based markers, often known as molecular markers, which exhibit greater versatility. Classical markers encompass morphological, cytological, and biochemical markers, while DNA markers include a variety of types such as Restriction Fragment Length Polymorphism (RFLP), Random Amplified Polymorphic DNA (RAPD), Amplified Fragment Length Polymorphism (AFLP), Simple Sequence Repeats (SSRs), Single Nucleotide Polymorphism (SNP) markers etc. [60]. In modern plant breeding, SNPs are extensively utilized as DNA markers to pinpoint genomic regions associated with key traits, thereby accelerating the breeding process. Recognized as the most prevalent variations within plant genomes, SNPs are invaluable for high-resolution genotyping, offering the highest map precision.

Moreover, SNPs are both more efficient and cost-effective compared to other markers. Their popularity surged in the twenty-first century, largely due to genotyping by sequencing (GBS) technique advancements. Some other novel marker techniques, such as Intron Length Polymorphism (ILP), Diversity Array Technology (DArT), Penta-Primer Amplification Refractory Mutation System (PARMS), Inter small RNA polymorphism (iSNAP), etc., have been employed in plant breeding, which has enabled precise selection of desirable traits in plants, genetic diversity analysis, and accelerated breeding (Amiteye, 2021). They pinpoint the precise genetic differences connected to desirable qualities, making it possible to pick individuals with the best genomic profiles for breeding with accuracy and efficiency. Recent advances in genomics are producing new plant breeding methodologies and ways (e.g., association mapping, marker-assisted selection, genomic selection, genome editing, etc.).

Genomic resources for plant breeding encompass genetic markers, reference genomes, genomic and protein databases, transcriptomes, and gene expression profiles. These resources facilitate the identification of genes associated with desirable traits, understanding genetic diversity, and acceleration of breeding programs [112]**.** Key techniques include genome-wide association studies (GWAS), marker-assisted selection (MAS), and genomic selection (GS), which allow for precise trait selection and prediction of breeding outcomes. By integrating these genomic tools, plant breeders can improve crop yield, disease resistance, and stress tolerance, enhancing agricultural productivity and sustainability. The existing variability among crop species is utilized for plant breeding activities, which can also be generated through crossing or induced mutagenesis. In addition to the identification of genetic markers and the availability of published genomes, clustered regularly interspaced short palindromic repeats-associated protein 9 (CRISPR/Cas9) is promising for application to modern breeding and is a novel technology for genome editing in major crops [132]. CRISPR/Cas9-based directional breeding is highly efficient and saves more time than other breeding techniques that use genome editing. It further enables targeted genetic modifications, opening new avenues for crop improvement.Genomics approaches are beneficial when dealing with complex traits, as these traits usually have a multi-genic nature and a significant environmental influence [102]. Genomic tools provide genomic information and facilitate the detection of QTLs and the identification of existing favorable alleles of small effect, which have frequently remained unnoticed and have not been included in the gene pool used for breeding [102]. Genomic tools have revolutionized plant breeding by enabling more precise, efficient, and targeted approaches to developing new plant varieties through genome-wide association study, marker-assisted selection, genomic selection, and gene editing. In this review, we present and discuss the most relevant advances in the development of genomic tools and provide examples of applying these tools to plant breeding.

### Molecular markers: tool for the genetic analysis

Based on nucleotide sequence polymorphisms, molecular markers include insertions, deletions, point mutations, duplications, and translocations. They are ideal when codominant, evenly distributed, highly reproducible, and detect significant polymorphism. The first molecular marker technique, RFLP, was introduced by Botstein et al. [15]. RFLP, RAPD, AFLP, and Isozyme markers are first-generation molecular markers that have been developed and used in genetic analysis and plant breeding (Table 1). Advancements in molecular markers have significantly enhanced their efficiency, resolution, and application scope in genetic analysis and plant breeding **(**Table 2**)** [156]. These advancements can be broadly categorized into the development of new types of markers, improvements in marker technologies, and the integration of molecular markers with other genomic tools. Here are some key advancements:

**Table 1:** Salient features of major molecular marker

Characteristics	RFLP	RAPD	AFLP	SSR	SNP	DArT
Genome abundance	High	Very High	Very High	Medium	Very High	Very High
DNA Quantity Needed (ng)	10,000	20	500–1000	50	50	50–100
DNA Quality Prerequisites	High	High	High	Low	High	High
Type of probes/primers	Genomic DNA or cDNA clones with short, single/low copy sequences	Usually, ten bp random nucleotide	Specific sequence	Specific sequence	Allele specific-PCR primers	Microarray chip
Locus specificity	Yes	No	No	Yes	Yes	Yes
Type of polymorphism	Single nucleotide changes, InDels	Single nucleotide changes, InDels	Single nucleotide changes, InDels	Variations in the length of repeats	Single nucleotide changes, InDels	Single nucleotide polymorphisms at restriction sites
Level of Polymorphism	Medium	High	High	High	High	High
Inheritance	Codominant	Dominant	dominant	Codominant	Codominant	Dominant
PCR requirement	No	Yes	Yes	Yes	Yes	No
Radioactive detection	Usually yes	No	Usually yes	No	No	No
Visualization	Radioactive	Agarose gel	Agarose gel	Agarose gel	SNP-VISTA	Microarray
Reproducibility	High	Low	Intermediate	High	High	High
Cost	High	Less	High	High	Variable	Cheapest

**Table 2:** Principle of advance molecular markers and their use in crop improvement

Marker type	Principle	Pros	Cons	Suitability in Breeding Contexts	Reference
SSRs (Simple Sequence Repeats)	SSRs are short tandem repeat sequences in the genome. Variation in the number of repeats is detected through PCR and gel electrophoresis	- High polymorphism - Co-dominant inheritance - Cost-effective - High reproducibility	-Labor-intensive - Requires high-quality DNA - Limited genome coverage	Best suited for MAS, genetic diversity studies, population structure analysis, and evolutionary studies in low-resource breeding programs	Gupta et al. [55]
SNPs (Single Nucleotide Polymorphisms)	SNPs are single-base variations at specific genomic positions. They are identified through sequencing or microarray-based techniques	- Abundant across genomes - Co-dominant inheritance - High throughput - Automation-friendly	- High cost of initial setup - Low polymorphism in some species - Requires sequence information	Ideal for high-resolution GWAS, genomic selection, QTL mapping, and fine-mapping in advanced breeding programs	Kumar et al. [86]
DArT (Diversity Arrays Technology)	DArT identifies polymorphisms based on the hybridization of genome-wide fragments to a microarray. It does not require prior sequence knowledge	- No prior sequence knowledge required - Cost-effective - High-throughput - Detects both known and unknown polymorphisms	- Dominant markers (less useful for co-dominant traits) - Lower resolution than SNPs - Requires specialized equipment	Useful for genome-wide diversity assessment, QTL mapping, MAS in under-researched or non-model crops where genome sequences are unavailable	Cruz et al. [32]
PARMS (Penta-Primer Amplification Refractory Mutation System)	PARMS detects SNPs or other mutations via allele-specific primers and real-time PCR, distinguishing alleles by melting curve analysis	- High specificity - Cost-effective - Scalable for large populations - Fast and real-time detection	- Requires real-time PCR equipment - Lower multiplexing capability than sequencing - Moderate technical difficulty	Suitable for SNP validation, MAS, and high-throughput genotyping in breeding programs, especially for traits with known SNPs	Xu et al. [179]
iSNAP (Inter Small RNA Polymorphism)	iSNAP detects polymorphisms in small regulatory RNAs (sRNAs) that impact gene expression. These are amplified and analyzed using PCR-based methods	- Targets regulatory regions - Detects functional variations - Useful for traits controlled by gene regulation - Emerging tool	- Limited technology and availability - Requires bioinformatics expertise - Lower throughput	Applicable for studying gene regulation in stress tolerance, metabolic traits, and developing regulatory SNP markers in breeding programs	Pant et al. [121]
ILP (Intron Length Polymorphism)	ILP detects length variations in intronic regions between exons using PCR amplification, revealing intron-based polymorphisms	- Genome-specific - Detects rare variants - High specificity - Low cost	- Low polymorphism frequency - Limited to non-coding regions - Lower throughput than SNPs	Useful for comparative genomics, phylogenetic analysis, and specific MAS for traits associated with intron polymorphisms affecting gene expression in crops	[97, 98]

#### SSRs or microsatellites

Microsatellites, alternatively known as short tandem repeats (STRs) or simple sequence repeats (SSRs), are short DNA sequences with lengths typically ranging from one to six base pairs in contrast to minisatellites (VNTRs), which feature longer repeat sequences spanning from 11 to 60 base pairs [115]. Microsatellites are found throughout the genome, including in chloroplasts and mitochondria [125, 127]. Due to the different numbers of repeats present in these locations, SSRs exhibit high polymorphism that is simple to detect using polymerase chain reaction (PCR). Mismatches, recombination, mobile element transfer (retrotransposons), and DNA strand slippage are some of the mechanisms that contribute to the occurrence of SSRs. Common SSR motifs encompass mononucleotide (A, T), dinucleotide (AT, GA), trinucleotide (AGG), and tetranucleotide (AAAC) repeats. The creation of primers often uses flanking sequences that are conserved around SSRs. Developing SSR markers involves creating an SSR library, identifying specific microsatellites, designing primers in favorable regions, and conducting PCR. Banding patterns are then interpreted and evaluated for polymorphism. SSR markers are highly favored due to their codominant inheritance, abundance, allelic diversity, and ease of assessment via PCR with flanking primers. McCouch et al. [107] conducted a pivotal study on Simple Sequence Repeat (SSR) markers in rice, focusing on developing and mapping these markers to enhance genetic research. They identified numerous SSR loci across rice chromosomes and designed primers for their use. This comprehensive set of SSR markers has become instrumental in rice genetic studies, including linkage mapping, trait association, and breeding. SSR markers, are used in plant genetics for various applications. For example, in rice, SSR markers have been used to map QTLs related to drought resistance and yield enhancement [65]. In wheat, SSR markers help identify genetic diversity and select for traits like disease resistance and stress tolerance [174, 175]. A 2023 study on Brassica napus utilized 304 SSR markers to evaluate genetic diversity, uncovering a 76% polymorphism rate and pinpointing loci associated with oil content and disease resistance [173]. The research emphasized SSR markers’ effectiveness in detecting allelic variation and mapping important agricultural traits. Additionally, SSR markers showed cross-species transferability, proving valuable for identifying traits beneficial for breeding programs and conserving genetic diversity. SSR markers are crucial for crop improvement and understanding genetic variation [83].

#### Inter small RNA polymorphism (iSNAP)

Endogenous noncoding small RNAs, typically 20–24 nucleotides long and have important regulatory roles, are widely distributed in eukaryotic genomes [54]. These small RNAs offer a valuable resource for molecular marker development due to their conserved flanking sequences, enabling primer design for PCR-based fingerprinting. The Inter small RNA polymorphism (iSNAP) technique, pioneered by Gui et al. [54], capitalizes on this characteristic. To detect length polymorphisms brought on by insertions and deletions (InDels) within the small RNA pool, primer pairs flanking small RNAs are used to start PCR reactions. It is a noncoding, sequence-based marker system and is suitable for genotyping and genome mapping [3]. Unlike traditional markers that focus on coding sequences or microsatellites, iSNAP markers explore variations in non-coding regulatory regions. This opens up new possibilities for studying gene expression and complex traits influenced by small RNA pathways, including plant stress responses, development, and epigenetic regulation. iSNAP markers have functional relevance, as they are closely associated with gene regulatory mechanisms. This makes them particularly useful for marker-assisted selection (MAS), enabling the identification of traits governed by post-transcriptional gene regulation, such as disease resistance and stress tolerance. A recent case study by Zhang et al. [196] illustrates the application of iSNAP markers in identifying disease-resistant genes in tomatoes (Solanum lycopersicum). In this study, researchers focused on identifying polymorphisms in intergenic regions flanked by microRNAs (miRNAs) associated with defense responses. They developed a set of iSNAP markers and used them to screen tomato varieties for resistance to Phytophthora infestans, the pathogen responsible for late blight disease. In maize, iSNAP markers have been used to identify genetic loci associated with disease resistance and yield traits, enabling more efficient breeding [97, 98]. In wheat, they assist in mapping quantitative trait loci (QTLs) for drought tolerance, enhancing the development of resilient varieties [26].

#### Intron length polymorphism (ILP)

In eukaryotic genomes, introns are prevalent and found throughout various gene components. Due to their lower selective pressure, Introns exhibit greater variability compared to coding sequences, rendering them valuable as highly polymorphic genetic markers. Recently, researchers have focused on annotating and leveraging gene introns to create intron-length polymorphism (ILP) markers on a genome-wide scale. An Intron Length Polymorphism marker (ILP marker) is a genetic marker used in plant and animal breeding to identify variations in the length of introns—non-coding regions of a gene that are transcribed but not translated into proteins. These markers exploit natural differences in intron lengths among individuals or populations, aiding in genetic mapping, diversity studies, and breeding programs by distinguishing between different genotypes or assessing genetic diversity [7]. These markers have proven invaluable for large-scale genotyping in major food crop plants, such as rice [7, 170], wheat [147], and maize [94]. PCR, a widely used technique, conveniently detects ILP markers. The amplification of introns via PCR involves designing primers in flanking exons, a method known as exon-primed intron-crossing PCR (EPIC-PCR). Notably, exon sequences tend to be more evolutionarily conserved, enhancing the versatility of primers designed within exons compared to noncoding sequences. ILP markers are particularly advantageous when they target multiple insertions and deletions (InDels) within a single intron during amplification. This strategy significantly increases the likelihood of identifying genetic polymorphism. ILP markers are highly transferable across related species, allowing for comparative genomics and evolutionary studies [27]. For example, in cereals such as rice, maize, and wheat, ILPs have shown consistent results, making them invaluable for research across different species without needing species-specific markers. Liu et al. [99] developed intron length polymorphism (ILP) markers for plants, identifying 1507 ILP markers in Oryza sativa (rice). These markers were highly transferable across species and showed polymorphism rates of 85.3%. ILP markers proved useful for genetic diversity analysis and breeding applications in various crops. A recent study by Chen et al. [27] demonstrated the effectiveness of ILP markers in mapping drought tolerance in rice (Oryza sativa). The researchers used ILP markers derived from conserved intron regions of the Dehydration-Responsive Element-Binding (DREB) gene family. These markers were employed to screen a diverse set of rice germplasm, leading to the identification of several drought-tolerant varieties. The study showcased how ILP markers could be used to identify quantitative trait loci (QTLs) associated with drought tolerance. The QTLs identified were then validated across multiple rice populations, proving the reliability of ILPs in marker-assisted selection (MAS) for complex traits like drought resistance. These developments underscore the importance of introns and their applications in molecular genetics, enabling more effective crop breeding and resource management.

#### Single nucleotide polymorphism (SNP)

**Fig. 1:**
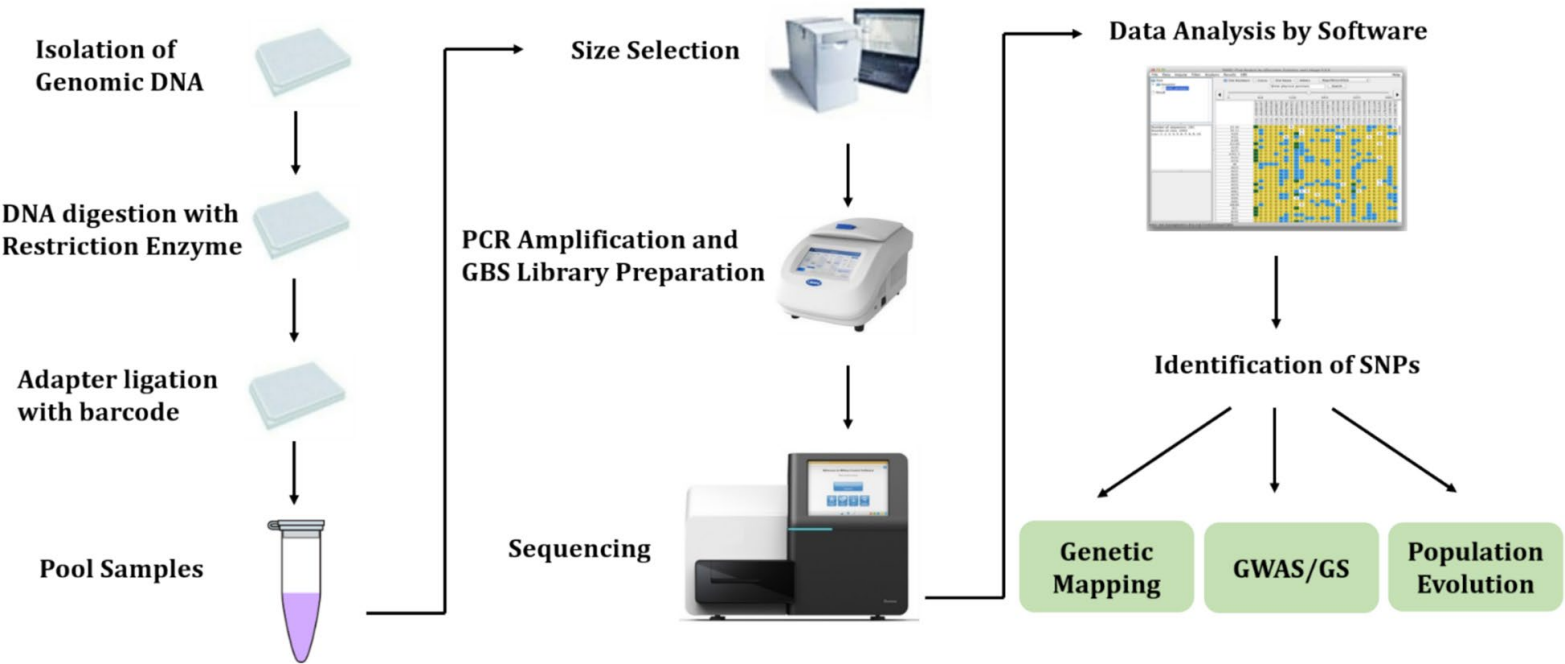
SNP discovery in plants through genotyping by sequencing (GBS) system and its application in crop improvement

Single Nucleotide Polymorphisms are single nucleotide differences seen in the genomic sequences of individuals within a population. These are the most prevalent molecular markers, and their distribution varies between species. Contrastingly, while humans exhibit an average of one Single Nucleotide Polymorphism (SNP) per every 1000 base pairs [137], rice displays a higher frequency, with approximately one SNP occurring within every 130–140 base pairs [51]. SNPs are frequently discovered in noncoding areas. According to Sunyaev et al. [158], SNPs in coding areas can be synonymous or nonsynonymous, changing phenotypic features and amino acid composition. SNPs are the smallest units of genetic variation, providing a simple and abundant source of markers crucial for genetic mapping, marker-assisted breeding, and map-based cloning. [188]. Significant techniques for SNP genotyping include primer extension, invasive cleavage, oligonucleotide ligation, and allele-specific hybridization [154]. SNP markers discovered by two methods, including SNP discovery from PCR and SNP discovery from High Throughput-Next generation sequencing (NGS) – RNA-Seq, RAD-Seq, Genotyping by Sequencing (Fig. 1), WGS (Whole-Genome Sequencing), WGR (Whole-Genome Regression), etc.

Next-Generation Sequencing (NGS) is a high-throughput DNA sequencing technology that enables rapid, parallel sequencing of millions of DNA fragments for comprehensive genomic analysis. In recent years, NGS technologies have identified thousands to millions of SNPs in various crops, facilitating genetic diversity studies, trait mapping, and breeding improvements. Numerous tools are available for SNP discovery, including BioEdit, DNASTAR Lasergene Genomics Suite, SAMtools, SOAPsnp, Stacks, Ddocent, PyRAD, and GATK. Typically, biallelic SNPs are straightforward to assay. SNP is detected when a nucleotide from an accession read differs from the reference genome at the corresponding position. Without a reference genome, this comparison is made by examining reads from different genotypes using de novo assembly methods. SNP calling is performed using read assembly files generated by mapping programs. Various empirical and statistical criteria, such as read depth, quality scores, and consensus base ratios, are employed in the SNP calling process. SNP discovery is more effective when multiple and diverse genotypes are analyzed simultaneously, as this approach captures the genetic variability within a species. There are three main types of SNP genotyping platforms: single SNP genotyping (using PCR with Taqman from Life Technologies or KASP genotyping from LGC Genomics), multiple SNP genotyping (using SNP chips from Illumina and multiplexing from Sequenom), and SNP genotyping by next-generation sequencing methods such as Genotyping by Sequencing (GBS) and Restriction site Associated DNA sequencing (RADSeq). For large-scale genotyping, high-throughput methods such as Genotyping by Sequencing (GBS), Restriction site- associated DNA sequencing (RADSeq), and allele-specific PCR are used [37]. These technologies have been extensively used to discover and genotype SNPs in food crops, including cereals [barley [31, 48, 134, 162], rice [28, 185], and wheat [20]], oil crops [oilseed rape [29] and sunflower [100]], horticultural crops [cowpea [30], potato [59], tomato [149]], soybean [155], and among others. SNP markers are crucial in genetics and agriculture, aiding genetic mapping, identifying disease associations, and improving crops through selective breeding. In their 2011 study, Kump et al. used SNPs to pinpoint disease resistance genes in maize, specifically targeting Southern Leaf Blight (SLB), caused by Cochliobolus heterostrophus. They identified 32 quantitative trait loci (QTLs) significantly associated with SLB resistance. The identified SNPs and associated QTLs can be used in marker-assisted selection (MAS) and genomic selection (GS) programs to develop SLB-resistant maize varieties. SNPs also play a pivotal role in integrating multi-omics data for crop improvement by acting as key genetic markers that link DNA variations to other molecular levels, such as gene expression (transcriptomics), protein abundance (proteomics), and metabolite profiles (metabolomics). These connections help to unravel the complex genetic architecture of important agronomic traits, including yield, stress tolerance, and disease resistance. By mapping SNPs to different omics layers, researchers can identify critical genes, pathways, and molecular interactions responsible for these traits [90]. This comprehensive approach enhances breeding accuracy, enabling the development of superior crop varieties with enhanced performance through more informed and precise selection methods.

#### Diversity array technology (DArT)

The DArT sequencing technique is a highly reproducible microarray-based method for discovering polymorphic markers [177]. DArT is a genomic analysis method designed to enhance the detection of SNPs (Single Nucleotide Polymorphisms) across the genome, particularly insertions and deletions. It begins with creating a purposefully randomized fragment library, which serves as a genomic representation. DArT libraries are tailored for specific research purposes, utilizing suitable individuals, whether individual or pooled samples. The subsequent steps involve identifying the genetic representations, hybridizing them onto the chips, and printing the genomic library onto microarray chips. DArT simplifies the genome by initially subjecting it to restriction digestion and then hybridizing the resulting DNA fragments onto microarray chips. Data analysis is done after scanning. This method allows thousands of genomic loci to be simultaneously genotyped in a single reaction test, requiring as little as 50–100 ng of genomic DNA. Once markers are identified, the need for specific assays for genotyping is eliminated, except for consolidating polymorphic markers into an array for a particular genotype. These genotyping arrays are equipped with these polymorphic markers and are commonly employed in genotyping tasks [66]. DArT markers are primarily dominant and require specialized software, laboratory facilities, a substantial investment, and skilled personnel (Sinha et al., 2023).

#### Penta-primer amplification refractory mutation system (PARMS)

The Penta-Primer Amplification Refractory Mutation System (PARMS) is a specialized genotyping technique used for identifying specific single nucleotide polymorphisms (SNPs) or mutations in DNA sequences. It is particularly useful in plant and animal genetics, as well as in medical research, for detecting alleles associated with certain traits or diseases. It is an extension of the traditional Amplification Refractory Mutation System (ARMS), which relies on the specificity of DNA primers to distinguish between different alleles at a given genetic locus [159]. PARMS involves the use of five primers to achieve high specificity and efficiency in identifying specific alleles. Two universal primers bind to conserved regions of DNA surrounding the SNP or mutation of interest. Two allele-specific forward primers and a reverse shared primer are designed to match perfectly with either the wild-type allele, mutant allele, or a third variant allele, with mismatches at critical positions near the SNP or mutation. This allows for selective amplification of only the specific alleles in question. It employs competitive allele-specific polymerase chain reaction (AS-PCR) and a fluorescence-based reporting system to detect genetic variations, specifically single-nucleotide polymorphisms (SNPs) [159]. It can efficiently handle different numbers of SNPs and samples to be analyzed. The process requires only standard liquid handling, thermal cycling instruments, and plate reading instruments.

Furthermore, it is compatible with DNA samples from various sources and extraction methods, including alkaline lysis. This makes it ideal for a direct PCR-based SNP marker-assisted selection system (D-MAS), known for its simplicity, cost-effectiveness, and labor efficiency in SNP genotyping. In a practical application, Gao et al. [49] developed a PARMS marker for the TAC1 gene, illustrating its usefulness in rice plant architecture breeding. Having outlined the significance of molecular markers, we now delve into the applications of genomic resources in crop improvement.

### Genomic resources for plant breeding

The availability of whole genome sequences is invaluable for plant breeding. Arabidopsis (125 Mb) and rice (466 Mb) were early models for plant genetics due to their small genomes among dicots and monocots. Their genome sequences, announced in 2000 and 2005, have been pivotal in understanding key genes and biological functions. The advent of next-generation sequencing (NGS) technologies has revolutionized genomics. Among these, the 454 (Roche) and Illumina platforms are widely used for crop sequencing. NGS technologies have significantly increased sequencing capacity; for instance, the Illumina HiSeq 2000 can generate 55 Gb per day, far exceeding the human genome size. The development of third-generation sequencing platforms like PacBio RS (Pacific Biosciences, https://www.pacb.com/), Helicos (Helicos, https://seqll.com/), and Ion Torrent has further advanced the field. These platforms enable the production of long reads, resulting in more accurate and contiguous genome assemblies. Third-generation sequencing is particularly effective for assembling genomes de novo, especially in regions with highly repetitive sequences and clarifying structural variants.

Additionally, isoform sequencing from these platforms facilitates detailed studies of exons, splice sites, and alternatively spliced regions, improving genome annotation. NGS-generated sequences are typically deposited in the NCBI Sequence Read Archive, making them accessible for further research. The emergence of third-generation sequencing has enabled the generation of long reads and allowed the production of more accurate and contiguous genome assemblies [25]. Third-generation sequencing enhances the creation of high-quality whole genome de novo assemblies by providing long reads that cover complex regions with highly repetitive sequences. This technology also elucidates other complex repeat sequences and structural variants. Third-generation sequencing techniques, such as isoform sequencing, produce full-length transcripts, enabling detailed analysis of exons, splice sites, and alternatively spliced regions, which aids in refining genome annotations. Sequences generated through NGS are typically archived in the NCBI Sequence Read Archive (https://www.ncbi.nlm.nih.gov/sra) for public access.

Two standard analyses performed on NGS reads are genome assembly and mapping. Assemblers like Roche's 454 Gsassembler, Celera Assembler, and Mira are frequently used for genome assembly. Once a reference genome is available, variation studies are typically conducted using mapping software such as Bowtie, BWA, and TopHat, which align reads to the reference genome. SNPs can then be detected with tools like SAMtools or GigaBayes. The algorithms for processing raw genomic data vary based on the data type and desired results. Bioinformaticians must present their findings to breeders via user-friendly interfaces, often through easily navigable websites. General-purpose web databases like GenBank (http://www.ncbi.nlm.nih.gov/genbank/), EMBL (http://www.ebi.ac.uk/embl/), DDBJ (http://www.ddbj.nig.ac.jp/), UniProt (http://www.uniprot.org), and Swiss-Prot (http://expasy.org/sprot/) provide researchers and breeders with essential biological information. Genomic sequence databases GenBank, EMBL, DDBJ, Ensembl, UCSC Genome Browser, and dbSNP offer extensive genomic data, analysis tools, and resources. Protein function databases, integrating sequence data, structural information, and functional annotations, include UniProt, Swiss-Prot, Gene Ontology, Protein Data Bank, InterPro, KEGG, Pfam, STRING, BioGRID, and PhosphoSitePlus (Table 3). Additionally, specialized databases for specific species useful to breeders, such as SGN, Phytozome, Gramene, and CropNet, provide targeted information for breeding programs. These resources collectively support the plant breeding process by enabling detailed genetic and protein analyses, aiding in developing improved crop varieties.

**Table 3:** Important Databases and Repositories of Genomic Information

Database	Description	URL
Genbank	General public sequence repository	http://www.ncbi.nlm.nih.gov/genbank/
EMBL	General public sequence repository	http://www.ebi.ac.uk/embl/
DDBJ	General public sequence repository	http://www.ddbj.nig.ac.jp
NCBI	Biomedical and genomic information	http://www.ncbi.nlm.nih.gov/
GOLD	Repository of genome databases	http://genomesonline.org/cgi-bin/GOLD/bin/gold.cgi
Phytozome	Genomic plant database	http://www.phytozome.net/
Plantgdb	Genomic plant database	http://www.plantgdb.org
CropNet	Genomic plant database	http://ukcrop.net/
CPGR	Phytopathogen genomic resource	http://cpgr.plantbiology.msu.edu/
Gramene	Cereals information resource	http://www.gramene.org/
R-PAN	Rice pan-genome browser for ~ 3000 rice genomes	http://cgm.sjtu.edu.cn/3kricedb/
Rice SNP-seek	A new SNP-calling pipeline	http://snp-seek.irri.org/
CerealsDB	Genotyping information for over 6000 wheat accessions	https://www.cerealsdb.uk.net/cerealgenomics/CerealsDB/indexNEW.php
SoyBase	Soybean information resource	http://soybase.org/
MaizeGDB	Maize information resource	http://www.maizegdb.org/
Tair	Arabidopsis information resource	http://www.arabidopsis.org/
SGN	Solanaceae information resource	http://solgenomics.net/
Gene Index Project	Transcriptome repository	http://compbio.dfci.harvard.edu/tgi/
UniProt	Protein sequences and functional information	http://www.uniprot.org/
Swiss-Prot	Protein Sequence database	https://www.expasy.org/resources/uniprotkb-swiss-prot
Protein Data Bank (PDB)	Repository for the 3D structural data of proteins and other biological macromolecules	https://www.rcsb.org/
Gene Ontology (GO)	Describe gene and protein functions across species and databases	https://geneontology.org/
InterPro	Integrates predictive models (known as signatures) from multiple databases into a single resource for protein classification	https://www.ebi.ac.uk/interpro/
KEGG	Annotations for genes and proteins, including their roles in pathways	https://www.genome.jp/kegg/
Pfam	Information on protein families and domains	http://pfam.xfam.org/
STRING	Database of known and predicted protein–protein interactions	https://string-db.org/
BioGRID	Database of protein and genetic interactions, chemical interactions, and post-translational modifications	https://thebiogrid.org/
PhosphoSitePlus	Comprehensive resource for the study of protein post-translational modifications	https://www.phosphosite.org/homeAction.action

New genomic tools are crucial for advancing and speeding up gene expression studies. Gene expression analysis provides breeders with valuable biological insights, helping them understand the molecular basis of complex plant processes and identify new targets for manipulation. While QRTPCR is an affordable, quantitative technique, it can only analyze a limited number of genes per experiment. Other methods, such as differential display and cDNA-AFLPs, allow the study of thousands of genes but lack quantitative precision and struggle with low-abundance transcripts (M Perez-de-Castro et al. 2012). More advanced techniques like serial analysis of gene expression (SAGE) and massively parallel signature sequencing (MPSS) address some of these limitations. However, the most popular methods today for transcript profiling are hybridization-based platforms or microarrays. Expression arrays offer several advantages, including measuring tens of thousands of transcripts simultaneously, semi-quantitative results, and sensitivity to low-abundance transcripts. Several web resources facilitate microarray data analysis, such as Babelomics (http://babelomics.bioinfo.cipf.es/), and software packages like Bioconductor (http://www.bioconductor.org/help/workflows/oligo-arrays/) and MeV (http://www.tm4.org/mev/) specialize in microarray analysis. Babelomics was used to analyze transcriptomic data in Arabidopsis thaliana to identify genes differentially expressed under drought stress. The tool facilitated functional annotation and identified key genes involved in stress responses [108] In Solanum tuberosum (potato), Bioconductor was utilized to analyze gene expression profiles under biotic stress conditions, identifying genes linked to pathogen resistance [194]**.** Genevestigator (https://www.genevestigator.com/gv/doc/plant_biotech.jsp) is a handy database containing extensive microarray data from various species, with the most comprehensive data from Arabidopsis thaliana. Data from crops like maize, wheat, rice, barley, and soybean are increasingly becoming available. Published expression data are publicly accessible in databases such as GEO (http://www.ncbi.nlm.nih.gov/geo/), ArrayExpress (http://www.ebi.ac.uk/arrayexpress/), and species-specific repositories, providing valuable resources for analyzing gene expression in these and other crops. A summary of genomic resources related to genome sequence and functional analysis is presented in Table 3.

### Genomic tools for plant breeding

Traditional plant breeding involves selection and crossing of plants with desirable traits over several generations. Techniques include selection of superior individuals, hybridization, and backcrossing. Breeders aim to enhance traits like yield, disease resistance, and quality. The process relies on natural genetic variation and careful observation to achieve desired improvements in crops over time. Genomic tools enhance traditional plant breeding by providing precise insights into genetic variations, speeding up trait identification, and enabling targeted modifications. They use DNA sequencing and markers to identify desirable traits more accurately and rapidly, reducing the time and cost of developing improved plant varieties compared to traditional methods that rely on broader, less precise selection processes [87]. These tools help identify desirable traits, speed up the breeding process, and improve the overall outcomes of breeding programs. Some key genomic tools in plant breeding include:

#### Quantitative trait Loci (QTL) mapping

QTL mapping is a statistical technique that combines phenotypic data (traits) with genotypic data (molecular markers) in a specific population to identify genetic regions (QTLs) associated with complex traits. Various QTL mapping models have been developed, such as standard interval mapping (SIM) and multiple imputation (IMP) for unlinked QTLs and composite interval mapping (CIM) for both linked and unlinked QTLs/genes on chromosomes. The effectiveness of these methods is evaluated using LOD (logarithms-of-odds) scores, with QTLs considered significant above a threshold LOD score of 3.0. Access to reference genomes provides valuable genetic information on QTLs, aiding marker-assisted selection (MAS). Traditional QTL mapping requires a balanced population with known recombination data, enabling statistical associations between phenotypic and genotypic data through linkage mapping (Lander et al. 1989). Identifying QTL locations helps pinpoint genes responsible for specific traits and understand genetic variation mechanisms [84, 105]. Several software tools facilitate efficient QTL mapping, including QTL Cartographer, QTL IciMapping, MapQTL, R/qtl, TASSEL, PLABQTL, and JoinMap. In the 2017 study by Duhnen et al., the accuracy of traditional QTL mapping was compared with newer genomic prediction models, such as GBLUP and Bayesian methods, in soybean. The results showed that genomic prediction models outperformed QTL mapping by utilizing genome-wide markers, capturing both major and minor effect loci. This led to higher predictive accuracy for complex traits like yield and seed protein content, making genomic selection more effective for breeding. QTL mapping bridges genomics and field studies by linking genetic regions to quantitative traits. However, integrating data from multiple QTL studies to identify high-quality candidate loci for crop breeding remains challenging. Meta-analysis, which combines results from various studies, is crucial in accurate QTL prediction. Tools like MetaQTL reduce the confidence interval of QTL, improving location and effect estimation. Other tools, such as solQTL and RASQUAL, offer low-bias QTL analysis and data visualization. Meta-QTL analyses have been applied to crops such as rice, wheat, maize, cotton, and soybean, identifying significant traits like yield-related genes in wheat [182] and nitrogen use efficiency QTLs in rice [82]. Van and McHale (2017) performed a meta-QTL analysis with genetic information comprised of 175 QTLs for protein, 205 QTLs for oil, 156 QTLs for amino acids, and 113 QTLs for fatty acids. They detected 55 meta-QTL for seed composition on 6 out of 20 chromosomes. They also identified candidate genes within each meta-QTL aiding in crop improvement program. While QTL mapping effectively links traits to genomic regions and detects rare alleles, its resolution is limited by parental allelic diversity. Genome-wide association studies (GWAS) can address these limitations by identifying trait-associated genomic areas of diverse populations.

#### Genome-wide association study (GWAS)

Like QTL mapping, genome-wide association studies (GWAS) employ statistical association mapping to link molecular markers with traits of interest. GWAS leverages diverse populations and historical recombination, addressing linkage disequilibrium (LD) caused by physical linkage, genetic drift, selection, and population structure [71]. This method assesses marker-trait associations in diverse populations, although it faces challenges with false positives due to population structure and kinship (Huang et al. 2014). To mitigate this, covariates for structure and kinship are incorporated into mixed linear models (MLMs). Various statistical tools are used for association mapping, including MLM, CMLM, ECMLM, MLMM, GLM, and FarmCPU. Among these, the FarmCPU model is particularly effective at controlling false positives [77]. With advancements in sequencing technologies, GWAS has become a prominent tool for identifying loci linked to natural trait variations in crops. A population needs to be genotyped once and can be repeatedly used to map different traits with new phenotypic data. Despite its high false positive rate due to population structures and genetic relationships, GWAS is favored for exploratory analyses to identify a wide range of genomic leads. Unlike QTL mapping, GWAS is more likely to pinpoint specific candidate genes for crop improvement. It has been applied to various crops, including rice, soybean, maize, wheat, and canola. [24, 33]. For example, in Oryza sativa indica, sequencing of 517 landraces identified approximately 3.6 million SNPs, with GWAS of 14 agronomic traits explaining over 36% of phenotypic variance [64]. Myles et al. [112] used genomic resources to conduct a genome-wide association study (GWAS) in grapevine, identifying 3,600 SNP markers. They discovered loci linked to key traits like berry color (VvmybA1) and powdery mildew resistance, showcasing the utility of GWAS in identifying agriculturally significant genes in crops. In maize, GWAS revealed the genetic architecture of leaf traits, linking variation in liguleless genes to upright leaves [160]. He et al. [61] integrated GWAS with transcriptomic data and identified candidate genes involved in yield-related traits. This integrative approach facilitates the functional validation of the candidate genes. Bioinformatics tools such as PLINK, which uses standard regression for genotype–phenotype associations [126], and TASSEL, which incorporates mixed linear models to account for population structure, enhance GWAS studies [16]. GAPIT, another advanced tool, efficiently handles large datasets with over 1 million SNPs in 10,000 individuals using compressed mixed linear models and model-based prediction and selection methods [93].

#### Genome-based molecular breeding

**Fig. 2:**
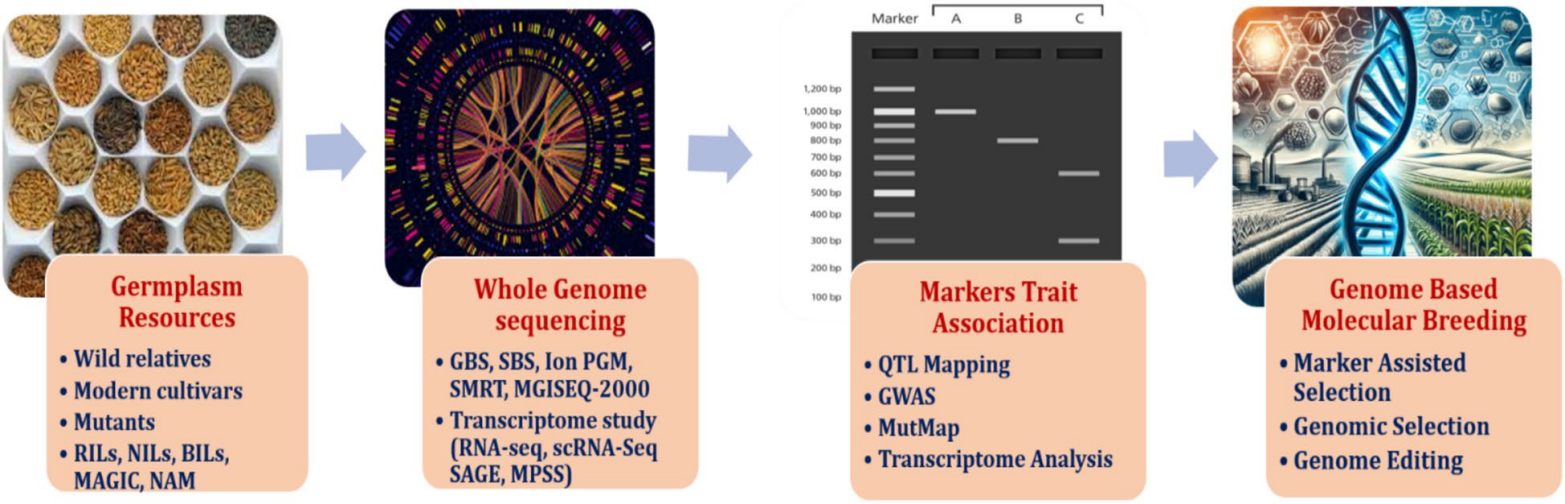
Trait discovery and genome based molecular breeding

Advancements in genomics and the availability of public genomic databases have significantly improved traditional breeding methods and facilitated the development of innovative crop breeding approaches (Fig. 2). Modern plant breeding focuses on evaluating the genetic gain of new genotypes by isolating genetic effects from environmental and noise components [12]. Different strategies, such as traditional phenotypic selection, marker-assisted selection (MAS), and genomic selection (GS), are employed. Traditional selection relies on phenotypic data for genetic evaluation. MAS, on the other hand, uses specific genetic markers linked to traits of interest, selecting individuals based on their marker scores [23]. Genomic selection is a more advanced method that considers markers with small effects on phenotypic variation. The process of genome-based molecular breeding involves several steps (Fig. 2). MAS, known for its efficiency, reduces both time and costs. MAS is faster as it doesn't require extensive phenotype testing of large progeny sets and allows for the pyramiding of multiple alleles.

Additionally, it reduces linkage drag and increases genetic gain compared to phenotypic selection [63]. Genetic merit can be evaluated in larger populations without losing genetic diversity. MAS-based breeding programs have been widely implemented in crops such as wheat, rice, maize, and tomato. For instance, MAS has been used to incorporate the Pi-ta gene into various rice varieties, enhancing resistance to rice blast disease caused by the fungus Magnaporthe oryzae [72]. Researchers identified the Sub1 gene in a flood-tolerant traditional rice variety called FR13A. The gene, located on chromosome 9 of the rice genome, was found to play a critical role in enhancing a plant’s ability to survive under submerged conditions. The Sub1 gene is part of a regulatory mechanism that involves three key ethylene response factors (ERFs)—Sub1A, Sub1B, and Sub1C. Of these, Sub1A is the primary gene responsible for submergence tolerance and enables rice plants to enter a state of metabolic dormancy, where growth and energy consumption are slowed, allowing the plant to conserve energy and survive for up to two weeks underwater. In 2009, researchers successfully integrated the Sub1 gene into several high-yielding, widely cultivated rice varieties, such as Swarna Sub1, IR64 Sub1, and Samba Mahsuri Sub1, using marker-assisted breeding techniques [144]. In wheat, Lr34 and Sr2 are two crucial genes that confer durable resistance to leaf rust and stem rust, respectively. These genes have been incorporated into wheat varieties using Marker-Assisted Selection (MAS) to develop improved cultivars viz. Avocet S (Lr34) and Thatcher (Sr2) that are resistant to rust diseases [184].

Similarly, the enhancement of oleic acid content in soybean oil using Marker-Assisted Selection (MAS) has been a significant achievement in soybean breeding. This improvement is primarily focused on selecting specific alleles at the FAD2-1A and FAD2-1B loci, which are critical in regulating the fatty acid composition of soybean oil [122]. The FAD2-1A and FAD2-1B genes encode the enzyme omega-6 desaturase, which is responsible for converting oleic acid (a monounsaturated fatty acid) into linoleic acid (a polyunsaturated fatty acid). By modifying or silencing these genes, the conversion of oleic acid to linoleic acid is reduced, leading to a higher proportion of oleic acid in the oil. Oleic acid is a healthier monounsaturated fatty acid that is more stable and resistant to oxidation than linoleic acid, which is more prone to rancidity. Through MAS, breeders have been able to select soybean plants carrying specific alleles at the FAD2-1A and FAD2-1B loci that reduce the activity of the FAD2 enzyme. Several high-oleic soybean varieties have been developed through this approach. These varieties contain over 70% oleic acid compared to traditional soybeans, which contain around 20–30%. Some of the notable improved varieties include: Plenish^®^, Vistive Gold^®^, and Monola^®^ [57]. MAS has been used in barley for yield-related and stress tolerance traits, resulting in elite lines with improved malting quality by transferring the thermostable α-amylase from wild barley into a commercial variety [178]. Next-generation sequencing (NGS) technologies have increased the number of available markers, significantly boosting breeding efficiency. Markers such as SSRs, SNPs, InDels, and haplotypes are now crucial for efficient genotyping and constructing genetic maps. For example, SSR markers were used to map the Als gene on the 3H chromosome of barley, which is associated with a low number of tillers [34]. In a study on barley, 83 significant marker-trait associations were identified for six yield-related traits under drought conditions [1]. Various strategies have been applied to identify QTLs for relevant traits in these crops, greatly enhancing genomic-based molecular breeding through MAS.

Genomic Selection (GS), developed by Meuwissen et al. in 2001, represents a significant advancement beyond conventional Marker-Assisted Selection (MAS). While MAS suits traits influenced by a limited number of major genes, it falters with quantitative traits, often governed by numerous minor genes, as seen in yield and stress tolerance traits. GS tackles this challenge by harnessing an array of genetic markers spread across the genome to compute Genomic Estimated Breeding Values (GEBV) for each individual. GEBV integrates phenotypic data with marker and pedigree data, yielding superior prediction accuracy compared to MAS [18, 62]. GS strategically selects genome-spanning genetic markers, ensuring that all Quantitative Trait Loci (QTLs) align with at least one marker [52]. The first step in the genomic selection process is creating a training population of individuals with genotypic and phenotypic data (Fig. 2). This data is used to construct a predictive model, employing phenotypes as responses and genotypes as predictors.

Insights derived from this model then enable the estimation of GEBV for the breeding population, consisting solely of individuals with genotypic data. Genomic Selection seems a viable approach to advance genetic development within breeding programs in climate change. Grain yield, qualitative characteristics, and abiotic stress resistance have all benefited from using genomic selection. This rapid improvement results from the selection of desired phenotypes across generations. Genomic selection offers a significant advantage by reducing breeding cycle duration and lowering phenotyping costs, thereby accelerating genetic improvements for food security. Factors influencing prediction accuracy include training and breeding population sizes, genetic diversity, heritability, genotype-environment interactions, marker density, and genetic relationships [96]. Several methods have evolved for genomic selection over time. An easy linear model, such as ordinary least-squares regression, is frequently used as the first step in the genomic selection process. An issue that frequently arises in linear models with a large number of genome-wide markers is that the number of markers (p) might significantly exceed the number of observations (n), leading to overparameterization. An alternative strategy is adapted to deal with this problem. A selection of significant markers is used in modified least-squares regression. This log-likelihood model may lead to the loss of important data when choosing markers with significant effects.

Ridge regression (RR) is a penalized regression technique that solves overparameterization and multicollinearity [109]. The least absolute shrinkage and selection operator (LASSO) is another variant, that may have difficulties with strongly correlated predictors [118]. Elastic net (ENET) is a modification of LASSO that balances the ℓ1 and ℓ2 penalties in LASSO and RR, respectively, and is robust to extreme correlations among the predictors [46]. A Bayesian method to estimate marker effects is provided by Bayesian models like Bayes A, Bayes B, Bayes C, and Bayes D [56]. Genomic BLUP (GBLUP), one of the best linear unbiased prediction (BLUP) techniques, uses genomic relationships to estimate genetic values [131]. Ridge regression (RR), best linear unbiased prediction (BLUP), least absolute shrinkage and selection operator (LASSO), support vector machine (SVM), artificial neural network (ANN), and random forest (RF) are examples of approaches based on single trait genomic selection (STGS). The multi-trait genomic selection technique (MTGS), which increases prediction accuracy for numerous characteristics, uses multivariate regression with covariance estimation (MRCE) and multivariate LASSO (MLASSO). A number of software tools and packages have been created to evaluate genomic prediction accuracy and make genomic selection (GS) usable. Some notable tools and approaches are rrBLUP, solGS, GMStool, BWGS, BGLR, GenSel, GSelection and lme4GS [18]. Genomic Selection (GS) revolutionizes crop improvement, streamlining breeding for precision and efficiency. Utilizing genetic markers, GS predicts crop yields (Table 4). For example, Cerioli et al. [21] used the LSU500 marker set to achieve predictive abilities of 0.13 to 0.78 in various rice trials. GS is equally potent in disease resistance breeding, as seen in Zhang et al.’s [189] study on Fusarium head blight (FHB) traits in wheat, yielding prediction accuracies of up to 0.59 for FHB disease index and 0.54 for Fusarium damaged Kernels (FDK). Furthermore, GS enhances abiotic stress tolerance. Zhang et al. [190] assessed maize drought tolerance with prediction accuracies ranging from 0.19 to 0.33 for traits like seedling emergence rate, plant height, and grain yield. Rutkoski et al. [136] reported that genomic selection (GS) improved wheat grain yield by 15% compared to conventional breeding methods. In their study, GS utilized 1,056 single nucleotide polymorphism (SNP) markers to predict breeding values, enabling more accurate selection and accelerating yield gains from 0.5% to 1.5% per year, demonstrating the effectiveness of GS in wheat improvement. In Maize two hybrids viz. Pioneer^®^ P1197AM and Pioneer^®^ P1329AM have been developed using genomic selection to enhance traits like yield and disease resistance [153]. GS facilitated the development of soybeans with enhanced resistance to diseases and pests, improving overall crop resilience and yield. Asgrow^®®^ variety of soybean has been developed through GS with improved disease resistance and enhanced yield potential [135]. These findings underscore Genomic Selection's potential to boost crop productivity and resilience to adverse environmental conditions and accelerates the development of new varieties with desirable traits. Recent advancements in plant breeding have prominently featured genome editing technologies which allows precise alterations to specific genes, enabling targeted modifications for traits like disease resistance or yield improvements. This approach provides exact changes at the genetic level. In contrast, genomic selection involves evaluating the genetic potential of plants using DNA markers linked to multiple traits. It assesses overall genetic merit and guides breeding decisions by predicting future performance. While editing is precise, selection offers a broader assessment for enhancing complex traits [114].

**Table 4:** Genomic prediction of traits in different crops

Crop	Model	Population Type	Trait	Prediction Accuracy	Reference
Rice	GBLUP	128 Japanese rice cultivars	Field grain weight	0.28	Yabe et al. [180]
			Variance of field grain	0.53	Yabe et al. [180]
	GBLUP, RKHS	Germplasm	Drought-resistance	0.23–0.80	Bhandari et al. [14]
	MT-RRM	357 Accessions	Daily water usage	0.29–0.87	Baba et al. [6]
			Projected shoot area	0.38–0.80	Baba et al. [6]
Wheat	GBLUP	F4:6 population	Grain Yield	0.75	Michel et al. [110]
	RRBLUP	Winter wheat breeding lines	Powdery mildew resistance	0.6	Sarinelli et al. [141]
	MLM	Winter wheat breeding lines	Stripe rust resistance	0.13–0.46	Shahinnia et al. [146]
Maize	GBLUP and multigroup GBLUP	TC	Grain Yield	0.78	Rio et al. [133]
	RRBLUP and GBLUP	Inbred lines and half diallel population	Water-logging tolerance	0.53–0.84	[35]
	GBLUP	Adapted and an exotic-derived maize population	Tocochromanols (vitamin E)	0.79	[161]
Soybean	RRBLUP	RILs from interspecific cross	Yield	0.68	Beche et al. [10]
	Extended Genomic BLUP	702 advanced breeding lines	Optimal cross combinations	0.56	Miller et al. [111]
Groundnut	Bayesian generalized linear regression	Breeding lines	Yield	0.49–0.60	Pandey et al. [120]
			Protein	0.41–0.46	Pandey et al. [120]
			Rust resistance	0.74–0.75	Pandey et al. [120]
Chickpea	RRBLUP	315 advanced breeding lines	Yield	0.33	Li et al. [90]
Lentil	RRBLUP	Diversity panel, RIL	Maturity duration	0.58–0.84	Haile et al. [58]

#### Genome editing

Genome editing, a powerful genetic modification method, enables precise alterations in DNA sequences using molecular scissors and artificial nucleases. Its origins date back to successful gene insertions in mammalian genomes in 1985–1986 [152]. Three main genome editing technologies are Zinc finger nucleases (ZFNs), transcription activator-like effector nucleases (TALENs), and CRISPR/Cas systems. CRISPR/Cas stands out for its simplicity, efficiency, and versatility [148]**.** Genome editing in agriculture enhances crop traits, like increased yield, disease and pest resistance, accelerating breeding programs, ensuring food security, and promoting sustainable farming practices in a rapidly changing world (Table 6). Clustered Regularly Interspaced Short Palindromic Repeats (CRISPR) system is an amazing bacterial defense mechanism against plasmid and virus invasion [75]. The sequences were first discovered in E. coli in 1987 when researchers found conserved direct-repeat sequences [69]. A crucial CRISPR/Cas9 development occurred in 2012 when Jinek et al. demonstrated SpCas9, derived from Streptococcus pyogenes, to be successful as an RNA-guided DNA endonuclease in vitro. This system inserts invading DNA pieces between crRNA repetitions in the host CRISPR locus. Mature crRNAs are produced through active transcription and pre-processing of these pre-crRNAs by Cas9 and host factors. Cas9 is directed to the appropriate target locus inside the invading DNA by the mature crRNA-Cas9 complex. The PAM motif (Protospacer adjacent motif) is often ahead of the site-specific cleavage that the Cas9 nuclease produces. The conventional PAM sequence 5' NGG 3' at the 3′ end of the target site is principally needed for the SpCas9. CrRNA and tracrRNA are combined to create chimeric single guide RNA (sgRNA), which directs Cas9 to the target region and introduces double-stranded breaks. Both homology-directed repair (HDR) and non-homologous end-joining (NHEJ) techniques can be used to fix these breaks. Customized gRNA-Cas9 complexes have been used to target economically significant traits in plants, highlighting the promise of CRISPR/Cas9 in agricultural applications [164].

In nature, there are two main types of CRISPR-Cas immune systems: class 1, which uses multiprotein complexes for nucleic acid cleavage, and class 2, which uses single-protein effector domains (Makarova et al., 2020). Because they benefit from a single protein effector, class 2 systems, especially types II, V, and VI, are preferred in biological research and translational applications. Regarding the class 2 Cas proteins, type-II Cas9 and type-V Cas12 are RNA-guided DNA endonucleases, whereas type-VI Cas13 mainly targets and cleaves RNA. Compared to type II CRISPR-Cas9, type V CRISPR-Cas12a (formerly CRISPR-Cpf1, a CRISPR from Prevotella and Francisella 1) and CRISPR-Cas12b are significantly different (Wang et al. 2020). SpCas9 requires a 5′-NGG-3′ PAM, whereas Cpf1 uses a 5′-TTTN-3′ or 5′-TTN-3′ PAM, hence extending the range of target sites in the genome. Additionally, Cas12a permits gene targeting with shorter crRNAs, possibly lowering genome editing costs [8]. The RNA-guided endonucleases known as Cas9 from type-II CRISPR systems have revolutionized genome editing. In the target DNA sequences, they cause double-strand breaks (DSBs) [73]. To create ribonucleoprotein complexes in their natural environment, Cas9 nucleases rely on CRISPR RNAs (crRNAs) linked with trans-activating crRNAs (tracrRNAs) [36]. However, single guide RNAs (sgRNAs), which combine crRNA and tracrRNA into one molecule, are used in the majority of genome editing applications [75]. An important sequence is the protospacer adjacent motif (PAM), which is located 3' of the target DNA strand in opposition to the guide RNA. Cas9 nuclease typically generates blunt-ended DSBs 3 base pairs upstream of the PAM, although some Cas9 nucleases exhibit alternative cutting patterns [142]. After the Cas9-guide RNA complex binds to the corresponding PAM, the DNA is unwound to create an RNA•DNA heteroduplex with the target DNA strand [73]. From the PAM-proximal region of the protospacer to the PAM-distal end, the process moves in one direction. 'R-loop' formed by the non-target DNA strand is a single-stranded DNA structure that is exposed and accessible. Base editing and prime editing, two more recent genome editing techniques, take advantage of this property [5]. Cas9 undergoes conformational changes, activating its nuclease domains during R-loop formation. After activation, Cas9 hydrolyses DNA's phosphodiester backbone via the HNH nuclease, cleaving the target DNA strand linked to the guide RNA and the RuvC-like nuclease, cleaving the non-target DNA strand containing PAM. Cas9 nickases, produced by mutations in either nuclease domain, are valuable for base and prime editing as they cleave a single DNA strand [130]. Both domains can be inactivated to create catalytically dead Cas9 (dCas9), retaining DNA binding abilities for applications like transcriptional regulation and epigenetic modifications. Many Cas9 variants have evolved since the discovery of Streptococcus pyogenes (SpCas9) Cas9 nuclease, varying in size, PAM recognition, guide RNA architecture, spacer length, editing efficiency, and specificity. SpCas9, the most popular, contains 1,368 amino acids, recognizes NGG PAMs, and supports both sgRNAs and crRNA/tracrRNA pairings with 20-nt spacers. However, it exhibits a higher off-target editing rate. Specialized Cas9 variants like SaCas9 (smaller, 1053 amino acids) and Nme2Cas9 (recognizing pyrimidine-rich PAMs) offer unique advantages, enabling researchers to tailor their choice of Cas9 effector for specific genome editing needs [42, 128]. This diversity advances the study of molecular biology and biotechnology by enabling researchers to choose the best Cas9 effector for their particular needs in genome editing. Wang et al. [169] reported a new gain-of-function OsGS2/GRF4 allele generated by CRISPR/Cas9 genome editing increases rice grain size and yield. Errum et al. [43] found that CRISPR/Cas9 editing of wheat Ppd-1 gene homoeologs alters spike architecture and grain morphometric traits and increases 1000-grain weight, grain width, grain length, plant height, and spikelets per spike. Genome editing has led to the development of several crop varieties with improved traits, showcasing the potential of this technology in agriculture. In rice, CRISPR-edited varieties such as Sasakure and Nipponbare have been developed to enhance yield and boost resistance to diseases, addressing key agricultural challenges [91]. In wheat, CRISPR-edited lines resistant to wheat blast provide protection against this devastating fungal pathogen, improving crop resilience and productivity [174, 175]. Wheat variety developed for drought tolerance through genome editing is the CRISPR/Cas9-edited wheat targeting the *TaDREB2* gene, which is associated with drought stress response. By modifying this gene, researchers improved the plant’s drought tolerance without negatively impacting yield. For instance, Kim et al. [79] reported enhanced drought tolerance in wheat through CRISPR/Cas9 by targeting transcription factors like *TaDREB2*, aiding in the development of more resilient wheat varieties in drought-prone regions. Similarly, in soybeans, genome editing has led to the creation of high oleic acid varieties, which improve the nutritional quality and stability of soybean oil, offering health and market benefits (Zhai et al., 2020). Alternative genome manipulation techniques such as base editing and prime editing offer distinct approaches for precise DNA modifications without causing double-strand breaks, beyond traditional methods like CRISPR-Cas9, making them ideal for crop improvement (Table 5). Base editing enables direct base conversion, such as converting cytosine to thymine. For example, it has been used to create herbicide-resistant rice by modifying the ALS gene [191, 195]. Prime editing is more versatile, allowing targeted insertions, deletions, and base substitutions. It has been applied to improve disease resistance in crops like rice by precisely altering genes like OsSWEET for bacterial blight resistance [191, 195]. Beyond simply modifying DNA sequences, CRISPR-Cas9 has also been adapted to control gene expression. Cas9 variants that lack cutting activity, such as dCas9 (dead Cas9), can be fused with transcriptional activators or repressors to modulate gene expression, opening new doors for research into gene regulation, epigenetics, and functional genomics. CRISPR-dCas9 offers a non-invasive way to control gene expression without introducing mutations. This is particularly advantageous in crops where regulatory and public concerns over genetically modified organisms (GMOs) are prevalent. By modulating gene activity instead of editing the genome, CRISPR-dCas9 allows for crop improvement while potentially avoiding some regulatory hurdles associated with traditional genetic modification. In a significant study by Lowder et al. [101], researchers used CRISPR-dCas9 technology to enhance yield and drought tolerance in tomato (Solanum lycopersicum). The researchers designed a CRISPR-dCas9 system where dCas9 was fused with transcriptional activators to upregulate genes involved in auxin biosynthesis and drought stress response. This enabled precise modulation of key genes without altering the tomato genome. A detailed study of genome editing has been presented in Table 6.

**Table 5:** Alternative genome editing systems

Genome editing	Concept	Application	Reference
Base editors	It is created by combining the single-stranded DNA deaminase enzyme with dormant SpCas9 (dSpCas9), which cannot create DSBs. Base editors precisely install targeted point mutations without the need for donor DNA templates, DSBs, or HDR. Adenine base editors (ABEs) and cytosine base editors (CBEs), which transform A•T to G•C pairings and C•G to T•A pairs, respectively, are the two main subcategories of base editors	A novel selectable marker for wheat and the development of herbicide tolerance traits; Development of herbicide-tolerant rice germplasm	Zhang et al. [192]; Kuang et al. [80]
CRISPR-associated transposases	It is an engineered Cas-transposon system that combines transposase with dCas9 for programmable DNA transposition. It efficiently inserts large genomic cargos into TA motifs in the genome. However, it has limitations, including off-target cargo integration and applicability only to bacterial cells	Type V-K CRISPR-associated transposases that are specifically designed to allow for precise cut-and-paste DNA insertion	Tou et al. [163]
Prime editors	It utilizes a specialized protein called Prime Editing Protein (PEP) and a prime editing guide RNA (pegRNA) with an extension containing the desired genetic edit. PEP introduces single-strand breaks in the DNA and copies the edit from the RNA extension into the genome, offering greater accuracy and versatility compared to traditional methods like CRISPR-Cas9	Correction of phenotypes and mutations in adult mice with hepatic and ocular disorders; Deletion and replacement of long genomic sequences	Jang et al. [70]; Jiang et al. [74]

**Table 6:** The application of genome editing techniques for the improvement of agronomic traits in crops

Crop	Technology	Target genes	Result/Trait improvement	Reference
Rice	CRISPR-Cas9	*GS2*	Increased grain size and yield and bigger grain length/width ratio	Wang et al. [169]
Rice	CRISPR-Cas9	*RBL1*	Broad-spectrum disease resistance	Sha et al. [145]
Rice	CRISPR-Cas9	*OsCul3a*	Xanthomonas oryzae/Magnaportheoryzae resistance	Gao et al. [50]
Rice	CRISPR-Cas9	*OsPi21, OsXa13*	Magnaportheoryzae/Xanthomonas oryzae resistance	Li et al. [89]
Rice	CRISPR-Cas9	*SWEET11, SWEET13 and SWEET14/promoter*	Xanthomonas oryzae pv. Oryzae resistance	Oliva et al. [119]
Rice	CRISPR-Cas9	*EBEs of OsSWEET14*	Resistance to Xanthomonas oryzaepv. oryzae	Zafar et al. [187]
Rice	CRISPR-Cas9	*ERA1*	Drought tolerance	Ogata et al. [117]
Rice	CRISPR-Cas9	*bHLH024*	Salinity tolerance	Alam et al. [2]
Rice	CRISPR-Cas9	*miR535*	Tolerance to drought and salinity	Yue et al. [186]
Wheat	CRISPR-Cas9	*Ppd-1*	Increase in 1000-grain weight, grain width, grain length, plant height, and spikelets per spike	Errum et al. [43]
Wheat	TALEN	*TaMLO*	Powdery mildew resistance	Wang et al. [172]
Wheat	CRISPR-Cas9	*TaNFXL1*	Fusarium graminearum resistance	Brauer et al. [17]
Wheat	CRISPR-Cas9	*NAC071-A*	Drought sensitive	Mao et al. [106]
Wheat	CRISPR-Cas9	*MYBL1*	Drought sensitive	Mao et al. [106]
Maize	ZFN	*PAT*	Herbicide resistance	Schornack et al. [143]
Maize	CRISPR-Cas9	*ZmPLA1*	Haploid induction in tropical maize line	Rangari et al. [129]
Maize	CRISPR-Cas9	*abh2*	Drought tolerance	[95]
Maize	CRISPR-Cas9	*STL1*	Salinity tolerance	Wang et al. [171]
Barley	CRISPR-Cas9	*HvMorc1*	Resistance against Blumeriagraminis and Fusarium graminearum	Kumar et al. [81]
Barley	CRISPR-Cas9	*HVP10*	Salinity sensitive	Fu et al. [47]
Barley	CRISPR-Cas9	*ß-1-3glucanase*	Reduction of callose deposition in maize sieve tubes	Kim et al. [78]
Arabidopsis	CRISPR-Cas9	*eIF4E*	Transgene free resistant against Clover yellow vein virus	Bastet et al. [9]
Arabidopsis	CRISPR-Cas9	*TRE1*	Drought tolerance	Nunez-Munoz et al. [116]
Arabidopsis	CRISPR-Cas9	*C/VIF1*	Salinity tolerance	Yang et al. [181]
Soybean	CRISPR-Cas9	*FAD2*	Increase in oleic acid content	Zhou et al. [197]
Soybean	CRISPR-Cas9	*GmF3H1/2, FNSII-1*	Soybean mosaic virus	[191, 195]
Soybean	CRISPR-Cas9	*MYB118*	Sensitive to drought, salinity	Du et al. [39]
Cotton	CRISPR-Cas9	*GhDIR5 and GhDIR6*	Toxicity-depleted cotton seed	Lin et al. [92]
Cotton	CRISPR-Cas9	*Gh14-3-3d*	Verticillium dahlia resistance	Zhang et al. [193]
Cotton	CRISPR-Cas9	*ALARP*	Cotton fiber development	Sander and Joung [139]
Tobacco	CRISPR-Cas9	*IR, C1*	Cotton leaf curl multan virus resistance	Yin et al. [183]
Tomato	CRISPR-Cas9	*Solyc08g075770*	Fusarium oxysporum f. sp. lycopersici	Prihatna et al. [123]
Tomato	CRISPR-Cas9	*GID1a*	Drought tolerance	Illouz-Eliaz et al. [68]
Tomato	CRISPR-Cas9	*ABIG1*	Salinity tolerance	Ding et al. [38]
Tomato	CRISPR-Cas9	*GRXS14, GRXS15, GRXS16, GRXS17*	Sensitive to heat, chilling, drought, heavy metal toxicity, nutrient deficiency	Kakeshpour et al. [76]
Brassica napus	CRISPR-Cas9	*BnCRT1a*	Verticillium longisporum resistance	Probsting et al. [124]
Potato	TALEN	*ALS*	Herbicide resistance	Butler et al. [19]
Capsicum	CRISPR-Cas9	*CaERF28*	Anthracnose disease resistance	Mishra et al. [113]

### Regulatory framework for genome-edited crops

Genome editing raises several ethical issues, including the possibility of unintended genetic changes, long-term effects on ecosystems, and disparities in access to technology. Potential risks involve off-target mutations and unforeseen consequences that could impact environmental stability or human health. Ethical discussions focus on maintaining responsible research practices, obtaining informed consent, and considering the broader societal impacts of genetic alterations. To manage these concerns effectively, it is vital to establish strong regulatory frameworks and foster open, transparent public discussions on the use of genome editing technologies [140]. Regulations for genome-edited crops vary globally [157]. In the USA, the regulatory framework for genome-edited crops is managed by three agencies. In the United States, the regulatory framework for genome-edited crops is relatively permissive. The U.S. Department of Agriculture (USDA) oversees genetically engineered (GE) plants under the Plant Protection Act. In 2020, the USDA announced that crops developed through genome editing, such as those edited with CRISPR/Cas9, would not be regulated if they do not contain DNA from other species. This approach is exemplified by the CRISPR-edited mushroom developed by Penn State researchers, which resists browning and was not subjected to USDA regulations because it does not introduce foreign genes [13]. However, genome-edited crops may still be subject to regulation by the Environmental Protection Agency (EPA) and the Food and Drug Administration (FDA), depending on their traits. The FDA reviews crops for food safety, while the EPA regulates plants engineered to produce pesticides. The European Union (EU) adopts a more stringent regulatory approach. Genome-edited crops are treated similarly to genetically modified organisms (GMOs) under EU regulations. This requires extensive safety assessments, risk evaluations, and labeling before they can be marketed. In 2018, the European Court of Justice (ECJ) ruled that genome-edited crops should be regulated under the same laws as GMOs, meaning they must undergo rigorous assessments [41]. For example, CRISPR-edited wheat developed by Rothamsted Research, aimed at improving disease resistance, faced substantial regulatory hurdles due to the EU’s stringent GMO regulations [11]. The wheat’s developers had to navigate a complex approval process, reflecting the EU’s cautious stance on genome editing. Canada’s regulatory framework is focused on the end product rather than the method of development. The Canadian Food Inspection Agency (CFIA) evaluates whether genome-edited crops require regulation based on their traits. If the modifications do not result in novel traits or substances, the crop may not require extensive oversight [22]. For example, CRISPR-edited canola with improved oil composition has been developed, and the CFIA determined that it did not necessitate extensive regulatory scrutiny as long as it did not introduce novel traits [151].

Australia’s approach is also product-based, with a focus on the characteristics of the final crop. The Gene Technology Regulator (GTR) assesses whether genome-edited crops contain new traits or genes that would warrant regulation. If the modifications do not introduce new genetic material or traits associated with GMOs, the crop may not face stringent controls [53]. An example is CRISPR-edited barley with enhanced disease resistance. The GTR decided that the crop did not require additional regulatory measures as long as the edits did not involve new genetic material [45]. Japan’s regulatory framework is evolving as the country seeks to balance safety with innovation. Initially, genome-edited crops were assessed under the existing GMO framework, which involves rigorous safety evaluations. However, Japan is working towards creating a more tailored approach to genome editing [103]. For example, CRISPR-edited soybeans with improved nutritional properties are currently under review. Recent discussions in Japan are focused on developing regulations that distinguish between genome editing and traditional GMOs, potentially streamlining the approval process [138]. The government of India issued an Office Memorandum on 30th March 2022 and exempted SDN-1 and SDN-2 genome-edited plants without foreign DNA from Rules 7 to 11 of the EPA, 1986. This streamlines approval, bypassing the Genetic Engineering Appraisal Committee (GEAC) for genome-edited crops in India. The global regulatory landscape for genome-edited crops varies widely, reflecting diverse national priorities and approaches to biotechnology. At present, the United States and India employs a permissive approach focused on the end product, while the European Union maintains strict GMO-like regulations. Canada and Australia use a product-based perspective, assessing crops based on their traits rather than their development method. Japan is evolving towards a more nuanced regulatory framework. These differences highlight the ongoing debate over balancing innovation with safety and environmental considerations in the era of advanced genetic engineering [138].

### Conclusion and perspectives

The future of plant breeding promises advancements through genetic tools, precision breeding techniques, and climate-resilient crops to address global food security. Key genomic resources include genetic markers, reference genomes, databases, transcriptomes, and gene expression profiles. These tools are crucial for identifying genes linked to desirable traits, understanding genetic diversity, and accelerating breeding programs. Molecular markers will continue to enhance traditional breeding by enabling the selection of disease-resistant, drought-tolerant, and high-yield plants, leading to faster and more precise crop development. Genomic selection is poised to revolutionize plant breeding. It harnesses big data and advanced analytics to predict an individual plant's performance based on its genetic makeup [18]. This enables breeders to select and propagate plants with the highest genetic potential, significantly shortening breeding cycles and increasing the efficiency of trait improvement. Genome editing technologies, like CRISPR-Cas9, offer unprecedented precision in crop enhancement. They allow for the targeted modification of specific genes to introduce or enhance desirable traits while minimizing unintended changes [4]. CRISPR/Cas-9 and related systems (such as CRISPR/Cas-12 and CRISPR/Cas-13) are recognized as groundbreaking tools for genome editing due to their precision, efficiency, affordability, and simplicity. These technologies facilitate highly accurate modifications of nuclear genomes. However, a significant challenge is the potential for off-target mutations, which can lead to harmful phenotypes and limit the broader application of genome editing. To address this issue, new CRISPR/Cas variants are being developed, and existing systems are being enhanced to minimize off-target effects by selecting specific single guide RNAs (sgRNAs) with fewer predicted off-target sites based on a comprehensive reference genome sequence. Genome editing holds immense potential for creating crops with improved nutritional content, reduced susceptibility to pests and diseases, and increased environmental resilience. Collectively, these tools are instrumental in developing crop varieties that meet the challenges of a growing global population, changing climates, and sustainable agriculture practices. However, ethical, regulatory, and safety considerations will shape their future deployment.

## Data Availability

Not applicable.

## Abbreviations

SNP: Single Nucleotide Polymorphism
CRISPR/Cas9: Clustered Regularly Interspaced Short Palindromic Repeats-Associated Protein 9
QTL: Quantitative Trait Loci
RFLP: Restriction Fragment Length Polymorphism
RAPD: Random Amplified Polymorphic DNA
AFLP: Amplified Fragment Length Polymorphism
SSR: Simple Sequence Repeats
STR: Short Tandem Repeats
VNTR: Variable Number of Tandem Repeats
PCR: Polymerase Chain Reaction
NGS: Next Generation Sequencing
NCBI: National Center for Biotechnology Information
EMBL: European Molecular Biology Laboratory
DDBJ: DNA Data Bank of Japan
UniProt: Universal Protein Resource
UCSC: University of California Santa Cruz
KEGG: Kyoto Encyclopedia of Genes and Genomes
STRING: Search Tool for the Retrieval of Interacting Genes/Proteins
BioGRID: Biological General Repository for Interaction Datasets
SGN: Sol Genomics Network
QRTPCR: Quantitative Real-Time PCR
